# Efficacy and safety of dupilumab in patients with moderate-to-severe atopic dermatitis and comorbid allergic rhinitis

**DOI:** 10.3389/fmed.2025.1556769

**Published:** 2025-04-16

**Authors:** Tengfei Tian, Yueguang Li, Guangmei Yuan, Wenxiu Jiang

**Affiliations:** ^1^Department of Otolaryngology, Eye & ENT Hospital, Fudan University, Shanghai, China; ^2^Department of Burns and Plastic Surgery, Zhongda Hospital, Southeast University, Nanjing, China; ^3^Department of Dermatology, Zhongda Hospital, Southeast University, Nanjing, China; ^4^Department of Otolaryngology-Head and Neck Surgery, The Second Hospital of Shandong University, Jinan, China

**Keywords:** atopic dermatitis, allergic rhinitis, dupilumab, comorbidity, type 2 inflammation

## Abstract

**Objective:**

To examine the efficacy and safety of dupilumab treatment in moderate-to-severe atopic dermatitis (AD) patients with comorbid allergic rhinitis (AR).

**Methods:**

A total of 26 patients received subcutaneous dupilumab every 2 weeks and were followed up for 16 weeks. The efficacy assessment was evaluated based on clinical parameters every 4 weeks, including the Eczema Area and Severity Index (EASI), Pruritus Numeric Rating Scale (P-NRS), Severity Index (EASI), and Dermatology Life Quality Scale (DLQI) for the severity of AD symptoms, and the total nasal symptom scores (TNSS), visual analogue scale (VAS) and Mini Rhino-conjunctivitis Quality of Life Questionnaire (MiniRQLQ) for the severity of AR symptoms. The serum total immunoglobulin E (IgE) and eosinophil levels were collected at baseline and 16 weeks after dupilumab treatment. Treatment-emergent adverse events (TEAEs) ware conducted to evaluate the safety profile of dupilumab.

**Results:**

Dupilumab treatment resulted in a significant improvement in AD symptoms after 16 weeks as demonstrated by reduced EASI, NRS, DLQI, and ADCT scores. In addition, dupilumab treatment had a significant improvement in AR symptoms based on reduced TNSS, VAS and MiniRQLQ scores. After 16 weeks of dupilumab treatment, the blood eosinophil count, eosinophil percentage, and total serum IgE levels were significantly decreased when compared with the baseline values (*P* < 0.01 both). During the treatment period, the incidence of TEAEs was 13.77% (*n* = 8). The most commonly reported TEAEs were injection-site reactions (*n* = 13, 60.25%),

**Conclusion:**

Dupilumab treatment resulted in clinically relevant improvements in symptoms of AD and AR and had an acceptable safety profile.

## Introduction

Allergic diseases are a heterogeneous group of allergen-induced immunological disorder, and often chronic and characterized by type 2 inflammation (1). The prevalence of allergic diseases, including atopic dermatitis (AD) and allergic rhinitis (AR) has increased dramatically worldwide in the past several decades and have emerged as a significant global health concern (2, 3). The pathophysiology of AD is complex and multifactorial, involving genetic disorders, skin barrier dysfunction, aberrant immune responses, and skin microbial dysbiosis (3, 4). For most mild-to-moderate severity AD patients, topical therapy with corticosteroids or calcineurin-inhibitors is sufficient (5). However, a long-term alternative to systemic therapy is required for patients with moderate-to-severe AD (6). AD is also strongly associated with the development of AR (7). 15%–61% of AD patients complicate with AR (8). AR is an IgE mediated inflammation of the nasal mucosa following exposure to allergens and characterized by symptoms such as nasal obstruction, rhinorrhea, itchy nose, and/or sneezing (2, 9). While the majority of AR patients can be effectively managed with antihistamines, topical corticosteroids, and allergen-specific immunotherapy, there remains a subset of severe cases that are inadequately controlled (9, 10). However, allergic symptoms may worsen when the AD and AR conditions coexist (11). The presence of allergic comorbidities in patients should be considered when selecting an appropriate systemic treatment strategy. The prospect of utilizing a single agent to address both AD and AR presents an enticing possibility.

In recent years, the development of monoclonal antibody (mAb)-based therapies against type 2 cytokines has represented a significant advance in the treatment for these allergic diseases (6). Therefore, the prospect of utilizing a single agent to treat both diseases AD and AR is notably promising.

Dupilumab, a fully human mAb, functions by inhibiting the IL-4 and IL-13 signaling pathways through blocking the IL-4R receptor alpha and IL-13 receptor alpha-1 subunits (12, 13). It has received approval from the US Food and Drug Administration for the treatment of patients with type 2 inflammatory diseases. It is indicated for the treatment of inadequately controlled, moderate-to-severe AD in patients aged 12 years and older in the United States, and in adult patients in the European Union (14). Patients treated with dupilumab demonstrated rapid and significant improvements across all evaluated measures of atopic dermatitis disease activity (6, 14). Furthermore, studies administering dupilumab have shown a reduction allergen-specific IgE levels and an improvement in allergic symptoms in sensitized AR patients (15). Overall, utilizing a targeted immunomodulator such as dupilumab enables systemic treatment, potentially addressing atopic and allergic conditions that often occur concomitantly.

Although studies have confirmed the efficacy of dupilumab in treating AD and AR independently, there are no systematically evaluated its simultaneous effects on patient-reported outcomes (PROMs) across both diseases, which remains undetermined. Because of AD and AR frequently coexist, the impact of shared IL-4/IL-13 blockade on their symptoms remains uncharacterized. This study aimed to assess the efficacy and safety of dupilumab as monotherapy in moderate-to-severe AD with comorbid AR patients.

## Materials and methods

### Study design and patients

This is an observational study of dupilumab therapy in moderate-to-severe AD patients and combined AR for evaluation of the efficacy and safety of dupilumab. AD was diagnosed according to the criteria developed by Hannifin-Rajka standard (16) and the diagnosis of AR according to the ARIA guidelines (17). Patients receiving dupilumab therapy were enrolled at No.2 Hospital affiliated Shandong University and Zhongda hospital of Southeast University between June 2022 and August 2024. This study approved by the institutional review board or ethics committee at each trial site. We obtained written informed consent from all participants before commencing the study. A total of 26 participants received dupilumab and were evaluated after 16 weeks treatment. Demographic and clinical characteristics of the patients were recorded, shown in (Table 1).

**TABLE 1:** Demographic and baseline clinical characteristics of study participants.

Characteristic	*n* = 26
**Sex, *n* (%)**	
Male	19 (73.1)
Female	7 (26.9)
Age, years, mean ± SD	47.9 ± 10.6
Weight (kg), mean ± SD	74.2 ± 15.1
BMI (kg/m^2^), mean ± SD	26.1 ± 5.3
Duration of AD (years), mean ± SD	4.6 ± 1.7
Duration of AR (years), mean ± SD	3.8 ± 1.1
Serum total IgE levels at baseline >400 KU/L, *n* (%)	14 (53.8)
Serum EOS levels at baseline >0.5 × 10^9^ /L, *n* (%)	11 (42.3)
**Comorbidity, *n* (%)**	
Allergic conjunctivitis	5 (19.2)
Asthma	4 (15.4)
Food allergy	2 (7.7)
Hives	1 (3.8)
**Patients receiving prior systemic medications for AD, *n* (%)**	
Topical corticosteroids	23 (88.5)
Topical calcineurin inhibitors	20 (76.9)
Antihistamines	19 (73.1)
TCM	14 (53.8)
Cyclosporine	10 (38.5)
Methotrexate	10 (38.5)
Oral corticosteroids	5 (19.2)
Azathioprine	3 (11.5)
**Patients receiving prior systemic medications for AR, *n* (%)**	
Antihistamines	22 (84.6)
Intranasal corticosteroids	21 (80.8)
Leukotriene receptor antagonists	11 (42.3)
Intranasal decongestants	10 (38.5)
Intranasal anticholinergics	6 (23.1)

Data are presented as *n* (%) or mean ± SD unless otherwise indicated. AD, atopic dermatitis; AR, allergic rhinitis; BMI, body mass index; TCM, traditional Chinese medicine.

### Inclusion and exclusion criteria

We enrolled patients who fulfilled the following inclusion criteria. The inclusion criteria were as follows: (a) aged between 18 and 70 years old; (b) diagnosis of a moderate-to-severe AD with the indication for treatment with dupilumab established by the National Medical Products Administration. (c) Conforming to the concomitant presence of AR. (d) Positive results of skin prick test, and/ or positive serum specific IgE test for pollens.

Patients were excluded from the study if they met any of the following criteria: (a) treated with a systemic glucocorticoid within 4 weeks prior to recruitment; (b) participation in any clinical study within the 3 months prior to recruitment; (c) treated with allergen specific immunotherapy within last 3 years; (d) received other mAb treatment prior to recruitment; (e) pregnant, breast-feeding women; (f) patients with immunologic suppression, diabetes mellitus, autonomic neuropathy, coronary heart disease or hypertension.

### Dupilumab treatment

All patients received subcutaneous injections of dupilumab (Sanofi, France) as per the prescribed treatment regimen every 2 weeks. The initial dose administered was 600 mg, followed by subsequent doses of 300 mg administered every 2 weeks for 16 weeks. Follow up observations were evaluated every 4 weeks.

### Assessments

Efficacy endpoints were collected to assess disease severity and included the percentage of patients who achieved an IGA response of 0 (clear) or 1 (almost clear) with ≥2 point improvement (IGA 0/1); ≥50, ≥75, or ≥90% improvement in EASI (EASI-50, EASI-75, or EASI-90); ≥4-point improvement in Pruritus NRS (P-NRS 4); and Dermatology Life Quality Index (DLQI) of 0 or 1 with a baseline score of ≥2 (DLQI 0/1) from baseline at weeks 4, 8,12 and 16. The effect of dupilumab on rhinitis-associated nasal symptoms and quality of life was measured using the TNSS, VAS and MiniRQLQ. Additionally, the efficacy of dupilumab was assessed by monitoring changes in the aforementioned scoring systems. The levels of eosinophils and serum total IgE were measured at the initial presentation and at 16 weeks of dupilumab treatment. Safety of treatment was also assessed according to the TEAEs profile, which was monitored and recorded in daily questionnaire throughout the study. Treatment-emergent adverse events were recorded throughout the study (Table 2).

**TABLE 2:** Treatment-emergent adverse events during dupilumab treatment.

TEAEs, *n* (%)	All patients (*n* = 26)
Any TEAEs	10 (38.5)
Patients with ≥2 TEAE	5 (19.2)
Patients with any TEAEs leading to permanent discontinuation	0 (0)
**Common TEAEs reported**	
Injection-site reactions	9 (34.6)
Cough	5 (19.2)
Pyrexia	4 (15.4)
Nausea	4 (15.4)
Headache	2 (7.7)
Conjunctivitis	2 (7.7)
Oropharyngeal pain	1 (3.8)

All data are *n* (%) of patients, TEAE, treatment-emergent adverse event.

### Statistical analysis

All statistical analyses were performed using GraphPad Prism software 9.0 (GraphPad software, San Diego, USA). Values are reported as mean ± standard deviation (SD) or number (percentage) except where explicitly stated. Continuous data were compared with the two-sample *t*-test and categorical data with Fisher’s exact test. Non-normally distributed data were assessed using the Mann-Whitney *U*-test. We used ANOVA for repeated measures and Dunnett’s test for post-hoc analysis. A value of *P* < 0.05 was considered to be statistically significant.

## Results

### Patient baseline demographics and clinical characteristics

A total of 26 patients were included in the study, consisting of 19 male individuals (73.1%) and 7 female individuals (26.9%). Detailed demographic data and clinical characteristics of all patients were outlined in Table 1. The mean ages of patients were (47.9 ± 10.6) years. The mean weights were (74.2 ± 15.1) and mean body mass index (BMI) was (26.1 ± 5.3) Kg/m^2^. The mean history duration of AD was (4.6 ± 1.7) years and the mean history duration AR was (3.8 ± 1.1) years. Among these patients, 5 (19.2%) patients had allergic conjunctivitis, 4 (15.4%) patients had asthma, 2 (7.7%) patients had food allergy, and 1 (3.8%) patient had hives. Three patients (11.5%) had combined diseases involving two or more systems. Prior to dupilumab treatment, all patients received multiple medications. Topical corticosteroids (*n* = 23, 88.5%), topical calcineurin inhibitors (*n* = 20, 76.9%), antihistamines (*n* = 19, 73.1%), traditional Chinese medicine (TCM) (*n* = 14, 53.8%), cyclosporine (*n* = 10, 38.5%), methotrexate (*n* = 10, 38.5%), oral corticosteroids (*n* = 5, 19.2%), and azathioprine (*n* = 3, 11.5%) were used for AD control before the current study, respectively. The percentages of patients received antihistamines, intranasal corticosteroids, leukotriene receptor antagonists, intranasal decongestants, and intranasal antileukotrienes, and intranasal glucocorticoids for AR control were 22 (84.6%), 21 (80.8%), 11 (42.3%), 10 (38.5%), and 6 (23.1%) before the current study, respectively. Details of drug use were summarized in Table 2.

### Efficacy

**FIGURE 1:**
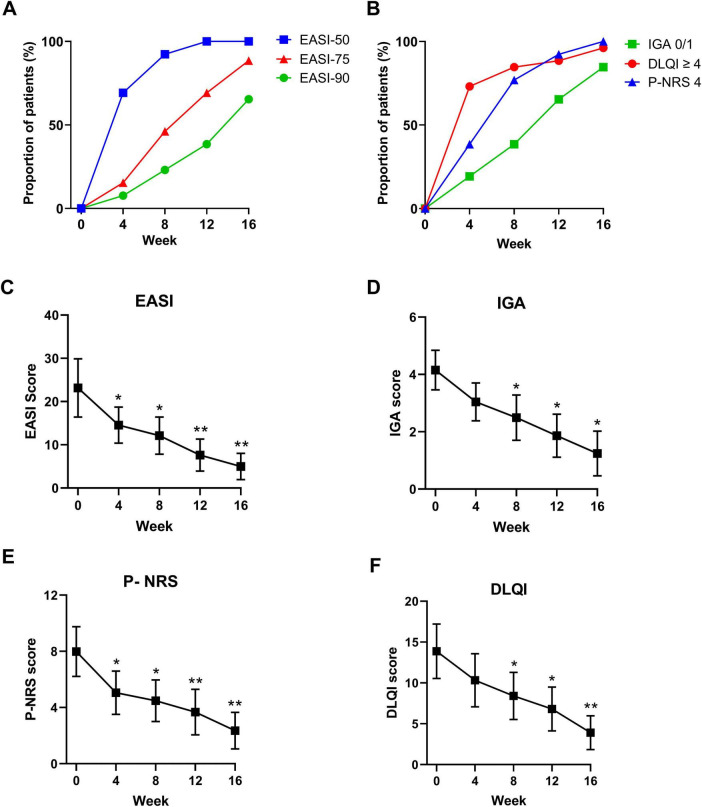
Dynamic change in the proportions of panel **(A)** EASI- 50, EASI-75, EASI-90, and achieved **(B)** IGA 0/1, P-NRS 4, and DLQI 0/1 at baseline, 4, 8, 12, and 16 weeks. Dynamic change in panel **(C)** EASI, **(D)** IGA 0/1, **(E)** P-NRS and **(F)** DLQI 0/1 at baseline, 4, 8, 12, and 16 weeks. EASI-50, 75, and 90, 50, 75, and 90% or more improvement in Eczema Area and Severity Index; IGA 0/1, Investigator’s Global Assessment score of 0 (clear) or 1 (almost clear); P-NRS 4, ≥4-point improvement in Peak Pruritus Numerical Rating Scale; DLQI 0/1, DLQI score of 0 or 1; *P < 0.05, **P < 0.01. Data are shown with mean ± SD.

In terms of efficacy of dupilumab for the treatment of AD symptoms, we analyzed severity measures such as EASI, IGA, measures of pruritus symptoms (pruritus NRS scores) and quality of life (DLQI scores). After 16 weeks of treatment, all patients (100%) achieved EASI-50, 23 (88.5%) patients achieved EASI-75, 17 (65.4%) patients achieved EASI-90, and 22 (84.6%) patients achieved IGA 0/1 (Figures 1A, B). The EASI score decreased from a mean of (23.1 ± 6.7) at baseline to (4.9 ± 3.0), and IGA score decreased from (4.2 ± 0.7) at baseline to (1.2 ± 0.8) (*P* < 0.001 both, Figures 1C, D). Furthermore, all patients had a decrease in pruritus NRS score of ≥4 (Figure 1B). The NRS score for pruritus decreased from (7.9 ± 1.8) at baseline to (2.4 ± 1.3) at the follow-up (*P* < 0.01, Figure 1E). Patients experienced a significant improvement in quality of life, as evidenced by a considerable decrease in DLQI scores, with 96.2% (25/26) patients showed a reduction of ≥4 points (Figure 1B). Additionally, The DLQI score decreased from (13.9 ± 3.3) at baseline to (3.9 ± 2.1) (*P* < 0.01, Figure 1F).

**FIGURE 2:**
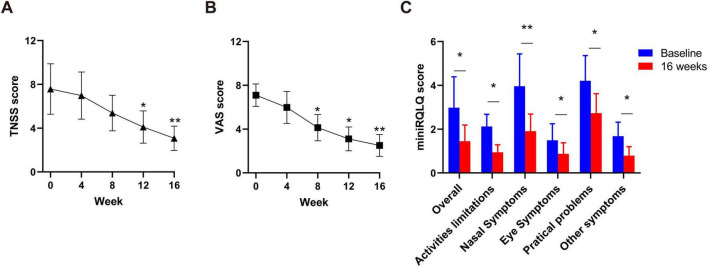
Dynamic changes of TNSS **(A)** and VAS **(B)** a at baseline, 4, 8, 12, and 16 weeks in all patients. Comparison of miniRQLQ scores **(C)** at baseline and week 16. VAS, Visual Analogue Scale; TNSS, Total nasal symptoms Score; miniRQLQ, Mini Rhino-conjunctivitis Quality of Life Questionnaire. *P < 0.05, **P < 0.01.

In terms of efficacy of dupilumab for the treatment of AR symptoms, we analyzed severity of AR symptoms (measured by the TNSS and VAS), and quality of life (measured by the miniRQLQ). After 16 weeks of dupilumab treatment, the TNSS scores were significant decreased from (7.6 ± 2.3) at baseline to (4.1 ± 1.5) and the VAS score decreased from (7.1 ± 1.0) at baseline to (3.1 ± 1.1) (*P* < 0.01 both, Figures 2A, B). Patients experienced significant improvements in all five domains of the miniRQLQ questionnaire. The overall miniRQLQ score significantly decreased from (2.9 ± 1.4) at baseline to (1.5 ± 0.7) (*P* < 0.01, Figure 2C).

### Effect of dupilumab on serum total IgE and eosinophil levels

**FIGURE 3:**
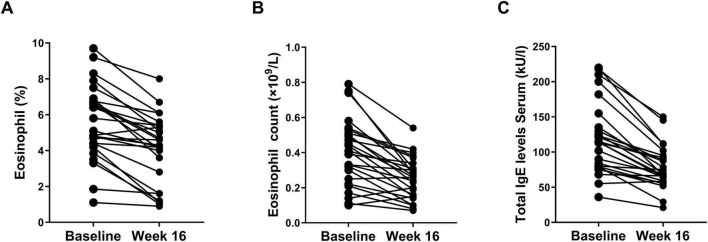
Changes of percentage of eosinophil **(A)**, eosinophil count **(B)**, and total IgE levels **(C)** at baseline and week 16.

All patients had their serum total IgE and eosinophil count levels compared baseline and at the 16-week follow-up. A total of 25 (96.2%) patients exhibited a decrease in total IgE levels, and 23 (88.5%) patients showing a decrease in eosinophil levels at 16 weeks (Figure 3). After 16 weeks of dupilumab treatment, the eosinophil count decreased from (0.40 ± 0.19) × 10^9^/L at baseline to (0.25 ± 0.12) × 10 ^9^/L, eosinophil percentage decreased from (5.60 ± 2.08)% at baseline to (4.12 ± 1.91)%, and the serum total IgE levels decreased from (119.2 ± 51.0) at baseline level to (77.8 ± 29.8) (*P* < 0.01 both, Figures 3A(C).

### Safety

Treatment-emergent adverse events were reported in 10 (38.5%) of the patients during the treatment period (Table 2). The most commonly reported TEAEs were injection-site reactions (*n* = 9, 34.6%), which presented as local itching, wind masses and pain at the injection site, but all of them were mild. In addition, cough, pyrexia, nausea, headache, oropharyngeal pain reactions and conjunctivitis occurred more frequently in patients were 5 (19.2%), 4 (15.4%), 4 (15.4%), 2 (7.7%), 2 (7.7%), and 1 (3.8%), respectively. Five patients (19.2%) had more than one TEAEs. No systemic AEs were observed among any of the patients receiving dupilumab. No patient discontinued study treatment owing to TEAEs. No deaths occurred during the study.

## Discussion

The results of this study show that dupilumab is effective in improving the signs and symptoms of moderate-to-severe AD patients with comorbid AR. This improvement manifested as reductions in both lesion extent and severity (EASI and IGA scores), alongside diminished pruritus (NRS scores) and demonstrably enhanced quality of life (DLQI) in AD control. Furthermore, the effect of dupilumab treatment on AR control were accompanied by significant improvements in AD control. These improvements manifested as alleviated nasal symptoms measured by TNSS and VAS scores, and improved quality of life measured by total miniRQLQ scores. Dupilumab showed good efficacy for both the skin lesions and allergic symptoms in AD patients with comorbid AR, which would increase the compliance and benefit of the patients (18). Moreover, dupilumab exhibited an acceptable safety profile throughout the study.

Dupilumab, an IL-4R antagonist that inhibits the IL-4 and IL-13 signaling pathway, has proven to be an efficacious inhibitor of type 2 inflammation (6, 13). Given the crucial role of Th2-mediated responses in type 2 inflammation driving allergic diseases, several clinical trials have evaluated the therapeutic potential of dupilumab (15). The efficacy and safety of dupilumab as monotherapy have been demonstrated in multiple clinical trials and real-world studies. These trials and studies have demonstrated promising clinical efficacy in various atopic and allergic diseases, including AD, AR, and asthma (15, 19). The significant influence of type 2 inflammation in moderate-to-severe AD manifests as a markedly elevated prevalence of allergic comorbidities. Because of their shared type 2 inflammatory mechanism, allergic comorbidities may increase disease burden and influence the management of patients with moderate-to-severe AD. A recent meta-analysis has demonstrated a significantly elevated prevalence of allergic comorbidities among AD patient (20). The analysis revealed that the overall pooled prevalence with concomitant AR, asthma and food allergy in AD patients was 40.5, 25.7, and 32.7%, respectively (20–22). All of which notably surpass the prevalence rates observed in individuals without AD. It has been widely acknowledged that AD and AR have similar immunopathological and functional characteristics, notably a predominant type 2 inflammatory profile (23). The comprehensive treatment effect of dupilumab across multiple aspects of AD patients with and those without AR may be due to the broad mechanism of action of dupilumab.

Within this profile, IL-4 and IL-13 play pivotal roles as central drivers, orchestrating immune cell recruitment and activation, disrupting epithelial barrier integrity, and contributing to microbiome dysbiosis (24, 25). By inhibiting IL-4 and IL-13 signaling through their shared receptor, IL-4 receptor alpha (26, 27), dupilumab effectively suppresses the type 2 inflammatory pathways underlying both AD and AR (28).

Similar effects of dupilumab on type 2 inflammatory biomarkers have been observed across clinical studies in multiple atopic and/or allergic diseases (29).

The coexistence of AD and AR significantly amplifies the overall disease burden, complicating disease management and often necessitating multiple treatment modalities. The demonstrated efficacy of dupilumab in treating atopic and allergic diseases presents a valuable opportunity to manage these coexisting conditions with a single therapeutic agent, offering a potential solution for this challenging patient population. Given the importance of Th2-mediated responses in type 2 inflammation in AD, several clinical trials have evaluated the role of dupilumab therapy. Consistent with previous studies and systematic review (6, 13, 21), our findings demonstrate that dupilumab treatment is associated with significant improvements in both the signs and symptoms of AD and overall quality of life.

The EASI assesses AD disease extent by considering erythema, induration/papulation, excoriation, and lichenification (21). IGA is an important indicator for clinical investigators to assess AD patients (30). After 16 weeks of dupilumab treatment, the EASI-75 and IGA 0/1 respondents were significantly improved. The EASI-75 response rate observed in our study (88.5% reduction) surpassed the previously reported range of 64%–75% in clinical trials focused on AD, demonstrating superior clinical efficacy (12, 31). Dupilumab treatment was also associated with a significant increase in the proportion of patients achieving IGA response (IGA 0/1). Pruritus, one of the most bothersome symptoms of AD patients (32). Achieving early control of pruritus is a key objective in the treatment of AD. In this study, with 85% patients demonstrated a ≥4-point improvement in pruritus NRS by week 16, which was higher than that reported in the standard loading dose group and previous clinical trial (33).

Dupilumab treatment led to a marked reduction in AR symptoms, as demonstrated by significant decreases in TNSS and VAS scores after 16 weeks. The benefits of treatment extend beyond mere improvement of AD symptoms, exerting other meaningful effects on the lives of patients. Similar to previous findings (34), our study demonstrated that nasal symptoms and quality of life also benefit greatly from dupilumab treatment. Previous study (15) showed the administration of 300 mg of dupilumab every 2 weeks resulted in TNSS scores reductions (mean: 5.03) in AR patients. In contrast, in our study showed accelerated nasal symptom relief and a more pronounced TNSS score reduction (mean: 4.1), suggesting enhanced therapeutic efficacy. These reductions in severity may also contribute to the significant improvements in quality of life observed in this study.

Quality of life considerations are an important element in assessing treatment response in AD patients. Previously, treatment with dupilumab was shown to be effective in preventing AD exacerbations and improving quality of life in patients with concomitant allergic asthma and persistent AR (35). In this study, we utilized the DLQI to monitor the impact of AD on various aspects of quality of life, including daily activities, personal relationships, symptoms and feelings, leisure activities, and work and school efficiency. In addition, we use the miniRQLQ to assess the impact of AR across five domains: activity limitations, practical problems and nose symptoms, eye symptoms and other symptoms. Our results showed that after dupilumab treatment, patients experienced a significant improvement in quality of life, as evidenced by a considerable decrease in DLQI and miniRQLQ scores, consistent with previous clinical trial. The superior AD outcomes may reflect synergistic effects of AR comorbidity management, as uncontrolled nasal inflammation exacerbates skin barrier dysfunction (36). Conversely, the accelerated nasal symptoms improvements could stem from dupilumab’s systemic modulation of Th2 pathways, which are shared across both diseases. Dupilumab has been shown to gradually suppress IgE levels when used as a monotherapy (37). Consistent with this fact, our data suggest a strong effect of dupilumab on reducing allergen-specific IgE levels, supporting its inhibitory effect of IL-4/IL-13 signaling leads to reduced serum IgE production and amelioration of allergic symptoms in sensitized patients. The observed progressive suppression of total IgE and eosinophil levels may also contribute to the gradual improvement in quality of life and nasal symptoms throughout 16 weeks treatment. In line with the established mechanism of action of dupilumab (8), its efficacy in patients with AD and comorbid AR was accompanied by significant reductions in both total serum IgE and eosinophil levels.

The incidence of total TEAEs was lower in the dupilumab group (38.5%) than previously reported results (77−78.6%) (6, 15, 38), indicating that dupilumab was well tolerated with an acceptable safety profile. Among the 26 patients, no new TEAEs were observed and associated with allergic comorbidities. Most TEAEs were mild or moderate. The safety profile of dupilumab in patients with comorbidities was generally manageable and consistent with that observed in the overall clinical study population.

Our study has some potential limitations. Firstly, the limited sample size posed challenges for analyzing the potential influence of various factors on the therapeutic efficacy of dupilumab across different patient subgroups. However, in future studies with a larger sample size, it is necessary to compare the efficacy and safety to provide clear evidence of dupilumab. Secondly, the follow-up time was only 16 weeks, so it may not be sufficient to confirm the long-term effectiveness and safety of dupilumab. Finally, this study recruited patients with moderate-to-severe AD whose condition was not controlled with topical medications, in accordance with the current approved indication for dupilumab. Consequently, the findings may not be generalizable to broader populations of AD patients with differing disease severity, phenotypes, or comorbidities. However, additional clinical studies are required to specifically interrogate the potential benefits of dupilumab in patients with AD coexisting AR.

## Conclusion

In conclusion, this study shows that treatment with dupilumab significantly improves the signs and symptoms, and improved quality of life in moderate-to-severe AD patients with comorbid AR. Moreover, dupilumab was generally well tolerated in this patient population. Collectively, dupilumab treatment has significant efficacy and safety in patients with AD and comorbid AR. Our findings indicate that dupilumab monotherapy not only effectively targets both AD and AR as isolated conditions, but may also exert a broader therapeutic effect through coordinated modulation multiple comorbidities of type 2 inflammation.

## Data Availability

The original contributions presented in this study are included in this article/supplementary material, further inquiries can be directed to the corresponding author.

## References

[1] Akdis C Arkwright P Brüggen M Busse W Gadina M Guttman-Yassky E Type 2 immunity in the skin and lungs. *Allergy.* (2020) 75:1582–605. 10.1111/all.14318 32319104

[2] Bousquet J Anto J Bachert C Baiardini I Bosnic-Anticevich S Walter Canonica G Allergic rhinitis. *Nat Rev Dis Primers.* (2020) 6:95. 10.1038/s41572-020-00227-0 33273461

[3] Ständer S. Atopic dermatitis. *N Engl J Med.* (2021) 384:1136–43. 10.1056/NEJMra2023911 33761208

[4] Hill D Spergel J. The atopic march: critical evidence and clinical relevance. *Ann Allergy Asthma Immunol.* (2018) 120:131–7. 10.1016/j.anai.2017.10.037 29413336 PMC5806141

[5] DaVeiga S. Epidemiology of atopic dermatitis: a review. *Allergy Asthma Proc.* (2012) 33:227–34. 10.2500/aap.2012.33.3569 22584191

[6] Beck L Thaçi D Hamilton J Graham N Bieber T Rocklin R Dupilumab treatment in adults with moderate-to-severe atopic dermatitis. *N Engl J Med.* (2014) 371:130–9. 10.1056/NEJMoa1314768 25006719

[7] Schneider L Hanifin J Boguniewicz M Eichenfield L Spergel J Dakovic R Study of the atopic march: development of atopic comorbidities. *Pediatr Dermatol.* (2016) 33:388–98. 10.1111/pde.12867 27273433 PMC5649252

[8] Davis D Drucker A Alikhan A Bercovitch L Cohen D Darr J American academy of dermatology guidelines: awareness of comorbidities associated with atopic dermatitis in adults. *J Am Acad Dermatol.* (2022) 86:1335–6.e18. 10.1016/j.jaad.2022.01.009 35085682

[9] Cheng L Chen J Fu Q He S Li H Liu Z Chinese society of allergy guidelines for diagnosis and treatment of allergic rhinitis. *Allergy Asthma Immunol Res.* (2018) 10:300–53. 10.4168/aair.2018.10.4.300 29949830 PMC6021586

[10] Meng Y Wang C Zhang L. Advances and novel developments in allergic rhinitis. *Allergy.* (2020) 75:3069–76. 10.1111/all.14586 32901931

[11] Silverberg J Gelfand J Margolis D Boguniewicz M Fonacier L Grayson M Association of atopic dermatitis with allergic, autoimmune, and cardiovascular comorbidities in US adults. *Ann Allergy Asthma Immunol* (2018) 121:604–12.e3. 10.1016/j.anai.2018.07.042 30092266 PMC13217624

[12] Wenzel S Castro M Corren J Maspero J Wang L Zhang B Dupilumab efficacy and safety in adults with uncontrolled persistent asthma despite use of medium-to-high-dose inhaled corticosteroids plus a long-acting β2 agonist: a randomised double-blind placebo-controlled pivotal phase 2b dose-ranging trial. *Lancet.* (2016) 388:31–44. 10.1016/S0140-6736(16)30307-5 27130691

[13] Wang F Tang X Wei C Xu L Mao H Luo F. Dupilumab treatment in moderate-to-severe atopic dermatitis: a systematic review and meta-analysis. *J Dermatol Sci.* (2018) 90:190–8. 10.1016/j.jdermsci.2018.01.016 29472119

[14] Gooderham M Hong H Eshtiaghi P Papp K. Dupilumab: a review of its use in the treatment of atopic dermatitis. *J Am Acad Dermatol.* (2018) 78:S28–36. 10.1016/j.jaad.2017.12.022 29471919

[15] Corren J Saini S Gagnon R Moss M Sussman G Jacobs J Short-term subcutaneous allergy immunotherapy and dupilumab are well tolerated in allergic rhinitis: a randomized trial. *J Asthma Allergy.* (2021) 14:1045–63. 10.2147/JAA.S318892 34429614 PMC8379710

[16] Hanifin J Tofte S. Update on therapy of atopic dermatitis. *J Allergy Clin Immunol.* (1999) 104:S123–5. 10.1016/s0091-6749(99)70054-0 10482863

[17] Brożek J Bousquet J Agache I Agarwal A Bachert C Bosnic-Anticevich S Allergic rhinitis and its impact on asthma (ARIA) guidelines-2016 revision. *J Allergy Clin Immunol.* (2017) 140:950–8. 10.1016/j.jaci.2017.03.050 28602936

[18] Bachert C Mannent L Naclerio R Mullol J Ferguson B Gevaert P Effect of subcutaneous dupilumab on nasal polyp burden in patients with chronic sinusitis and nasal polyposis: a randomized clinical trial. *JAMA.* (2016) 315:469–79. 10.1001/jama.2015.19330 26836729

[19] Blauvelt A Guttman-Yassky E Paller A Simpson E Cork M Weisman J Long-term efficacy and safety of dupilumab in adolescents with moderate-to-severe atopic dermatitis: results through week 52 from a phase III open-label extension Trial (LIBERTY AD PED-OLE). *Am J Clin Dermatol.* (2022) 23:365–83. 10.1007/s40257-022-00683-2 35567671 PMC9142443

[20] Knudgaard M Andreasen T Ravnborg N Bieber T Silverberg J Egeberg A Rhinitis prevalence and association with atopic dermatitis: a systematic review and meta-analysis. *Ann Allergy Asthma Immunol.* (2021) 127:49–56.e1. 10.1016/j.anai.2021.02.026 33684526

[21] Christensen M Barakji Y Loft N Khatib C Egeberg A Thomsen S Prevalence of and association between atopic dermatitis and food sensitivity, food allergy and challenge-proven food allergy: a systematic review and meta-analysis. *J Eur Acad Dermatol Venereol.* (2023) 37:984–1003. 10.1111/jdv.18919 36695076

[22] Ravnborg N Ambikaibalan D Agnihotri G Price S Rastogi S Patel K Prevalence of asthma in patients with atopic dermatitis: a systematic review and meta-analysis. *J Am Acad Dermatol.* (2021) 84:471–8. 10.1016/j.jaad.2020.02.055 32112994

[23] Tomassen P Vandeplas G Van Zele T Cardell L Arebro J Olze H Inflammatory endotypes of chronic rhinosinusitis based on cluster analysis of biomarkers. *J Allergy Clin Immunol.* (2016) 137:1449–56.e4. 10.1016/j.jaci.2015.12.1324 26949058

[24] Soyka M Wawrzyniak P Eiwegger T Holzmann D Treis A Wanke K Defective epithelial barrier in chronic rhinosinusitis: the regulation of tight junctions by IFN-γ and IL-4. *J Allergy Clin Immunol.* (2012) 130:1087–96.e10. 10.1016/j.jaci.2012.05.052 22840853

[25] Bachert C Claeys S Tomassen P van Zele T Zhang N. Rhinosinusitis and asthma: a link for asthma severity. *Curr Allergy Asthma Rep.* (2010) 10:194–201. 10.1007/s11882-010-0096-0 20424997

[26] Macdonald L Karow M Stevens S Auerbach W Poueymirou W Yasenchak J Precise and in situ genetic humanization of 6 Mb of mouse immunoglobulin genes. *Proc Natl Acad Sci U S A.* (2014) 111:5147–52. 10.1073/pnas.1323896111 24706858 PMC3986150

[27] Le Floc’h A Allinne J Nagashima K Scott G Birchard D Asrat S Dual blockade of IL-4 and IL-13 with dupilumab, an IL-4Rα antibody, is required to broadly inhibit type 2 inflammation. *Allergy.* (2020) 75:1188–204. 10.1111/all.14151 31838750 PMC7317958

[28] Harb H Chatila T. Mechanisms of dupilumab. *Clin Exp Allergy.* (2020) 50:5–14. 10.1111/cea.13491 31505066 PMC6930967

[29] Hirano I Dellon E Hamilton J Collins M Peterson K Chehade M Efficacy of dupilumab in a phase 2 randomized trial of adults with active eosinophilic esophagitis. *Gastroenterology.* (2020) 158:111–22.e10. 10.1053/j.gastro.2019.09.042 31593702

[30] Rehal B Armstrong A. Health outcome measures in atopic dermatitis: a systematic review of trends in disease severity and quality-of-life instruments 1985-2010. *PLoS One.* (2011) 6:e17520. 10.1371/journal.pone.0017520 21533286 PMC3076368

[31] Blauvelt A de Bruin-Weller M Gooderham M Cather J Weisman J Pariser D Long-term management of moderate-to-severe atopic dermatitis with dupilumab and concomitant topical corticosteroids (LIBERTY AD CHRONOS): a 1-year, randomised, double-blinded, placebo-controlled, phase 3 trial. *Lancet.* (2017) 389:2287–303. 10.1016/S0140-6736(17)31191-1 28478972

[32] Silverberg J Gelfand J Margolis D Boguniewicz M Fonacier L Grayson M Patient burden and quality of life in atopic dermatitis in US adults: a population-based cross-sectional study. *Ann Allergy Asthma Immunol.* (2018) 121:340–7. 10.1016/j.anai.2018.07.006 30025911

[33] Paller A Simpson E Siegfried E Cork M Wollenberg A Arkwright P Dupilumab in children aged 6 months to younger than 6 years with uncontrolled atopic dermatitis: a randomised, double-blind, placebo-controlled, phase 3 trial. *Lancet.* (2022) 400:908–19. 10.1016/S0140-6736(22)01539-2 36116481

[34] Campion N Doralt A Lupinek C Berger M Poglitsch K Brugger J Dupilumab reduces symptom burden in allergic rhinitis and suppresses allergen-specific IgE production. *Allergy.* (2023) 78:1687–91. 10.1111/all.15653 36691369

[35] Vignola A Humbert M Bousquet J Boulet L Hedgecock S Blogg M Efficacy and tolerability of anti-immunoglobulin E therapy with omalizumab in patients with concomitant allergic asthma and persistent allergic rhinitis: solar. *Allergy.* (2004) 59:709–17. 10.1111/j.1398-9995.2004.00550.x 15180757

[36] Geba G Li D Xu M Mohammadi K Attre R Ardeleanu M Attenuating the atopic march: meta-analysis of the dupilumab atopic dermatitis database for incident allergic events. *J Allergy Clin Immunol.* (2023) 151:756–66. 10.1016/j.jaci.2022.08.026 36084766

[37] Gandhi N Pirozzi G Graham N. Commonality of the IL-4/IL-13 pathway in atopic diseases. *Expert Rev Clin Immunol.* (2017) 13:425–37. 10.1080/1744666X.2017.1298443 28277826

[38] Weinstein S Katial R Jayawardena S Pirozzi G Staudinger H Eckert L Efficacy and safety of dupilumab in perennial allergic rhinitis and comorbid asthma. *J Allergy Clin Immunol.* (2018) 142:171–7.e1. 10.1016/j.jaci.2017.11.051 29355679

